# Determinants of Method Switching among Social Franchise Clients Who Discontinued the Use of Intrauterine Contraceptive Device

**DOI:** 10.1155/2015/941708

**Published:** 2015-10-20

**Authors:** Waqas Hameed, Syed Khurram Azmat, Moazzam Ali, Wajahat Hussain, Ghulam Mustafa, Muhammad Ishaque, Safdar Ali, Aftab Ahmed, Marleen Temmerman

**Affiliations:** ^1^Marie Stopes Society, Research, Monitoring & Evaluation Department-Technical Services, Karachi, Sindh 75500, Pakistan; ^2^Department of Uro-Gynecology, University of Ghent, 9000 East Flanders, Belgium; ^3^Department of Reproductive Health and Research, World Health Organization, 1211 Geneva, Switzerland

## Abstract

*Introduction*. Women who do not switch to alternate methods after contraceptive discontinuation, for reasons other than the desire to get pregnant or not needing it, are at obvious risk for unplanned pregnancies or unwanted births. This paper examines the factors that influence women to switch from Intrauterine Contraceptive Device (IUCD) to other methods instead of terminating contraceptive usage altogether. *Methods*. The data used for this study comes from a larger cross-sectional survey conducted in nine (9) randomly selected districts of Sindh and Punjab provinces of Pakistan, during January 2011. Using Stata 11.2, we analyzed data on 333 women, who reported the removal of IUCDs due to reasons other than the desire to get pregnant. *Results*. We found that 39.9% of the women do not switch to another method of contraception within one month after IUCD discontinuation. Use of contraception before IUCD insertion increases the odds for method switching by 2.26 times after removal. Similarly, postremoval follow-up by community health worker doubles (OR = 2.0) the chances of method switching. Compared with women who received free IUCD service (via voucher scheme), the method switching is 2.01 times higher among women who had paid for IUCD insertion. *Conclusion*. To increase the likelihood of method switching among IUCD discontinuers this study emphasizes the need for postremoval client counseling, follow-up by healthcare provider, improved choices to a wider range of contraceptives for poor clients, and user satisfaction.

## 1. Introduction

Contraceptive discontinuation is not uncommon, though the rates vary from country to country [1, 2]. On average, 38% of women discontinue using reversible method by the 12th month. The discontinuation of any modern contraceptive is 13% (IUCD) to 50% (condom) within the first 12 months of its use [3]. According to a report based on developing countries, 13.1% of IUCD users discontinue its use during the first 12 months, 26.3% within 24 months, and 36.7% by the third year of its use [2]. Research evidence shows that contraceptive users are less likely to discontinue the method for which they are required to visit a clinic or need assistance from health professionals such as IUCD and implants, compared to discontinuation of short-term or traditional methods [3–6].

Not all women who discontinue contraception become nonusers; some switch to other contraceptive methods [1]. It has been estimated that approximately half of the women that discontinue IUCDs switch to another method within a 3-month period [4] and about 35.6% switch to short-term contraceptive methods. This indicates that those who switch are likely to be highly motivated to restrict fertility whereas others may be intimidated by its side effects, which can be the main reason behind IUCD removal [7].

Women who do not switch to alternate methods after the discontinuation of IUCDs for reasons other than the desire to get pregnant are at risk of having unplanned pregnancies or unwanted births [8–10]. Also, method switching is indicative of strong family planning programs that have an adequate range of available methods coupled with a service environment flexible to women's needs [11]. Women when not satisfied with quality of care often consider method switching [12]. However, woman's age, parity, geographic location (urban versus rural), and education are the most consistent predictors of method switching [4, 13, 14].

High rates of contraceptive discontinuation (for reasons other than the desire to get pregnant) are highlighted as a public health concern due to their association with negative reproductive health outcomes [10]. It has been recommended that healthcare providers should be motivated to encourage women that had discontinued a method to opt for another method of their choice [15]. Discontinuation and low rates of switching to alternate methods have been neglected by family planning programs in many developing countries [5]. The increased contraceptive use in developing countries has cut the number of maternal deaths by 40% over the past 20 years, by merely reducing the number of unintended pregnancies. Increased contraceptive use has reduced the maternal mortality ratio by about 26% in little more than a decade; a further 30% of maternal deaths could be avoided by fulfillment of unmet need for contraception [16]. Yet, in order to understand the mechanisms through which contraceptive usage contributes to fertility decline, it is important to first understand and examine contraceptive dynamics, including contraceptive failure and method switching [17].

## 2. Rationale

Pakistan has a population of over 184 million [18] where 65% of the people live in rural areas [19]. Each year, over 12,000 women die due to preventable pregnancy-related complications [20] and nearly 2.2 million cases of induced abortions are reported [21, 22]. Modern contraceptive use is only 26% with a majority using either permanent or less effective methods, while the use of long-term methods is negligible (IUCD = 2.3% and implant is negligible). More specifically, the use of IUCD remains unchanged since 2006-2007 [23, 24]. The overall situation in the rural terrains of Pakistan is far worse with respect to the aforementioned health indicators. Generally, factors affecting method switching vary from country to country [6]. The latest national Demographic Health Survey (DHS 2012-13) reveals that overall 37% of contraceptive usage episodes are discontinued within 12 months for any particular reason, while side effects or health concerns are the most often cited reason for stopping use of the pill, IUCDs, and injection. Only 8% of IUCD users switch to another method [24].

Marie Stopes Society “*Suraj*” Social Franchise Network:* Suraj* Social Franchise (SF) is essentially a partnership between Marie Stopes Society (a nongovernment organization) and local health providers, aiming to increase demand, access, choices, and provision of quality family planning services in rural, underserved, and poor communities. The model uses a two-pronged approach: demand side and supply side. The demand side includes the provision of local area female health educators (FHEs) for raising awareness in communities regarding family planning and the referral of clients to service providers. Other components include free vouchers for long-term contraceptive method (IUCD) for the poor and comprehensive training of service providers on short-term (oral pill, condom, injectable, and emergency contraceptives) and long-term (IUCD insertion and removal) contraceptive methods and infection prevention.

The service providers mainly belong to midlevel providers category including lady health visitor, nurse, or community midwife. In each district the network ranged from 4 to 7 service providers practicing in the far-flung areas, each covering a total of 20,000–25,000 population.

There is very little evidence available in Pakistan with regard to this context. Therefore, in order to fill the gap in the existing body of knowledge, this paper reports on the cross-sectional data collected from women who received IUCD services from Marie Stopes Society's (MSS) Social Franchise providers in Pakistan, branded as “*Suraj*” (meaning “sun” in English) [15, 25].

This paper attempts to examine the factors that affect women's decision to switch to other contraceptive methods after the removal of IUCDs, instead of terminating usage altogether. The findings are also expected to be used for enhancing programme efficiency.

## 3. Methods

### 3.1. Data

The data used for this study come from a larger cross-sectional survey conducted by Marie Stopes Society in nine (9) randomly selected districts of Sindh and Punjab provinces during January 2011. The districts included Bahawalnagar, Jhang, Kasur, Lodhran, Sheikhupura, and Sialkot from Punjab and Umerkot, Hala/Matiari, and Tando Muhammad Khan from Sindh. The key objective of that survey was to estimate IUCD discontinuation rates and its determinants [15].

Participants were selected employing a multistage sampling with stratification. The first stage included district selection, the second stage included* Suraj* providers, and the final stage included selection of IUCD clients. The sampling details can be found elsewhere [15].

Women who received IUCD services (through* Suraj* providers) 6, 12, and 24 months prior to the survey were selected for this study. Women aged between 15 and 49 years and willing to give informed consent were invited to participate in the survey.

Face-to-face interviews were conducted with study participants using an adapted structured questionnaire that had previously been used in Philippines [26]. On an average, each interview took 20–25 minutes. The questionnaire covered sociodemographic characteristics (women's age, education, and number of living children), reasons for method discontinuation along with source of removal, switching behaviour, and client satisfaction the IUCD services. Data were double-entered in Visual FoxPro version 6.0 (Copyright © 1988–1998 Microsoft Corporation).

Out of 3,000 women, a total of 2,789 (93% response rate) women were successfully interviewed, of which 526 women had removed IUCD at some point in time before the survey due to any reason. We further eliminated cases where women discontinued the use of IUCD due to the desire for pregnancy and performed analysis on 333 cases for this paper.

The study protocol was reviewed and approved by the Research & Metrics Department of Marie Stopes International (MSI), London, UK.

## 4. Study Variables

The dependent variable was “method switching” where women coded “1” if they began to use another contraceptive method including traditional methods (within 1 month) after IUCD removal and “0” for women who stopped practicing contraception or became nonusers.

Some key independent variables included demographic characteristics (women's age, education, and number of living children), geographic region, type of IUCD, receiving IUCD through free vouchers, contraceptive status before the insertion of IUCD, experiencing method related side effects after IUCD insertion, reasons for IUCD insertion and removal, duration of IUCD use before discontinuation, time taken to travel to a* Suraj* facility, and the source of IUCD removal services.

## 5. Statistical Analysis

We analyzed data using Stata 11.2 (StataCorp. 2009, Stata Statistical Software: Release 11; StataCorp LP, College Station, TX). Simple frequencies and percentages were used to describe sample sociodemographic and health services characteristics. The association between explanatory variables and method switching was assessed using univariable and multivariable logistic regression techniques. A *P* value of ≤0.05 was taken to indicate statistical significance. The variables that showed a *P* value of >0.20 were not included in the multivariable modeling. Moreover, few satisfaction indicators (“recommendation of IUCD to friend” and “willingness to use IUCD in future”) were excluded from the final model due the issue of multicollinearity.

## 6. Results

Table 1 describes the characteristics of women who had removed IUCD due to any reason other than the desire for pregnancy. A majority (45.0%) of the respondents belonged to Southern Punjab, aged between 25 and 35 years (65.5%), and had no formal education (62.2%) and almost half had 5 or more children at the time of survey. Moreover, 62.8% had received IUCD through vouchers (for free), 76.6% had inserted the multiload, two-thirds were not using any form of contraception prior to IUCD insertion, and three-fourths had experienced method related side effects after IUCD insertion.

## 7. Switching Behaviors

The contraceptive status of women prior to IUCD insertion and after IUCD removal is presented in Table 2.

### 7.1. Overall Switching Method

Overall, within one month after the removal of IUCD, 2 out of 5 women abandoned the usage of contraception altogether whereas 33.3% and 19.8% opted for short-term and traditional methods, respectively.

### 7.2. Switching among Nonusers

Among women who were not using any method prior to IUCD insertion, a majority (45.2%) did not switch to other contraceptive methods and became nonusers after the removal of IUCD; 29.0% switched to short-term methods and 17.6% started using traditional methods.

### 7.3. Switching among Short-Term Contraceptive Users

Similarly, amongst women who were using short-term methods prior to IUCD insertion, a majority (44.9%) returned to short-term methods, 30.6% became nonusers, and 19.4% opted for traditional methods.

### 7.4. Switching among Traditional Method Users

Among users of traditional methods prior to IUCD insertion, 57.1% (*n* = 14) had returned to the same while 21.4% of women switched to short-term methods and an equal proportion became nonusers.

## 8. Univariate Analyses

The association between risk factors and method switching is presented in Table 3 by means of unadjusted odds ratios. Women living in Southern Punjab had 3.35 times higher odds of switching to another contraceptive method compared to women from Northern Punjab. Among health services variables, women practicing contraception before IUCD insertion were more likely to switch to another method as compared to those who were not using any contraception (odds ratio, 2.02). Similarly, users of multiload had 2.10 times higher odds of switching as compared to users of Copper-T. Moreover, women who received IUCD services for free (through voucher scheme) were less likely to switch to another method after its removal as compared to women who paid out of pocket for IUCD insertion. We also found that women who discontinued the use of IUCD within 3 months or between 3 and 6 months were more likely to switch to another method as compared to women who discontinued after 6 months of usage. Interaction or meeting with community health workers after IUCD removal substantially increased the chances of switching (odds ratio, 3.39). A measure of satisfaction levels also showed significant association with method switching.

## 9. Multivariable Analyses

In multivariable analyses fewer variables remained significant at a 5% level of significance (Table 4). Prior use of contraceptive methods and postremoval follow-up with community health worker showed positive association with method switching. Women who received IUCD for free (through voucher scheme) were less likely to switch to another method. Moreover, women who felt neutral or dissatisfied with IUCD were more likely to switch to another method. The women residing in Southern Punjab had 3.41 times higher odds of switching as compared to the women in Northern Punjab.

## 10. Discussion

The findings of this study reveal that only three-fifths of the women switched to another method after IUCD removal within one month, leaving others at right of unintended pregnancy at a given point in time [8–10]. Moreover, it is noteworthy to observe that women who were using any contraceptive method before the insertion of IUCD were more likely to switch back to the same (short-term) contraceptive methods after IUCD removal, which were less effective [27]. A possible reason for this may be attributed to the fact that the* Suraj* services providers were midlevel providers who are not allowed to provide implant that is another form of long-term contraceptive method or female sterilization (permanent method) as per national health policy. Yet, these services may have been available elsewhere.

The study also elicited higher chances of switching among women who were practicing contraception before the insertion of an IUCD. This behavior aligns with the aforementioned results (in Table 2) yielded from the study, depicting that women tend to revert to the original method that they were using before IUCD uptake. Also, keeping in view of the smaller difference between the proportion of ever and current use in Pakistan [23], it may be previous contraceptive exposure, experience, and henceforth knowledge that motivate women to switch to another method of their choice, instead of stopping usage altogether.

The study revealed that women who had received IUCD for free (through voucher scheme) were less likely to switch to another method, indicating that cost is a significant factor influencing method uptake after IUCD discontinuation [24]. It is also pertinent to note that the free voucher scheme only provided IUCD services whereas clients had to pay for the other modern contraceptive services irrespective of their economic status. Since vouchers were provided to clients that lacked affordability, they may have been restricted in terms of choice for alternate free modern contraceptive methods. Perhaps they would have preferred another method but were unable to afford it. However, there may be other influencing factors as poor clients can always switch to traditional methods. The findings warrant further investigation on this aspect for better understanding [28].

Moreover, women who met with community health workers after IUCD removal were more likely to adopt another contraceptive method. This is consistent with another study conducted in Bangladesh [1, 29]. It also emphasizes the need of repeated follow-ups and counseling to women after the removal of an IUCD, so that they may be better guided towards alternate contraceptive options.

We observed that users who were less satisfied with IUCD were more likely to switch to another method upon its removal which is consistent with earlier study where women who are less satisfied with quality of care often switch to another method [12]. This might have been due to higher satisfaction levels of women with previous contraceptive exposure that motivated women to revert to the same method.

Finally, our study also found different switching rates by geographic region. Though similar results were observed in other studies [17, 30], we suggest in-depth investigations to understand this phenomenon in our context. Interestingly enough, this study did not find association between method related side effects and method switching. This stands in stark opposition to the results from other studies conducted on the matter that depict IUCD side effects as one of the major reasons behind IUCD discontinuation [1, 3].

This study also has some limitations, common to all retrospective studies. A major limitation was the potential of recall bias, owing to the time lag between the client's IUCD discontinuation and when the survey was actually undertaken. The study only focused on women who discontinued the use of IUCD only whereas data regarding other contraceptive methods are beyond the scope of this study. Moreover, we did not capture data on partner-related factors which may influence the behaviour or practice of contraceptive use. Also, the data did not capture the continuation time of the new method uptake, post-IUCD discontinuation, among women who opted for an IUCD removal. This might have been a significant contribution in indicating whether new method was used for a day, a week, or few months and whether clients switched back to an IUCD after the new method uptake. Similarly, those who did not opt for a new method within a month of removal might have used any contraceptive method later on. Lastly, because the study is cross-sectional, deriving temporal associations is not possible. For example, it is assumed that the interaction/follow-up by a healthcare provider increases the likelihood of switching; the reverse may also be true; that is, the motive to switch causes the contact with healthcare provider. A comprehensive prospective study for a longer period of time could answer these questions.

Despite the aforementioned limitations, the insight generated from this study reveals interesting findings. To the best of authors' knowledge, this study is the first of its kind in Pakistan which specially focuses on contraceptive method switching behaviour and its determinants.

## 11. Conclusion

To promote method switching among IUCD discontinuers, this study emphasizes the need for effective counseling services and follow-up by community health workers. Immediate FP counseling and follow-up can significantly increase contraceptive uptake after IUCD removal especially among the women who discontinue a method for reasons other than the desire to get pregnant. Moreover, improved choices to a wider range of contraceptives for poor clients, quality services, and user satisfaction are crucial for promoting method switching in order to prevent the risk of unintended pregnancies. In addition, it will help dispelling myths and misconceptions regarding IUCD and may help in increasing the stagnated contraceptive use in the country. Yet, we recommend a rigorous prospective mix-method research to substantiate or endorse the determinants of method switching identified in our study.

## Figures and Tables

**Table 1:** Percentage distribution of women who removed IUCD due to any reason (other than pregnancy desire).

Characteristics	Women discontinued *n* (%)
Geographic region	
Sindh	59 (17.7)
Southern Punjab	150 (45.1)
Northern Punjab	124 (37.2)
Women received IUCD	
24 months ago	108 (32.4)
12 months ago	107 (32.1)
6 months ago	118 (35.4)
Age group of women	
≤25 years	21 (6.3)
>25–≤35 years	218 (65.5)
>35–49 years	94 (28.2)
Women's education	
No formal education	207 (62.2)
Primary	72 (21.6)
Secondary	45 (13.5)
Intermediate and post	9 (2.7)
Number of alive children	
1-2	47 (14.1)
3-4	120 (36.0)
5+	166 (49.9)
Type of client	
Referral (paid out of pocket)	124 (37.2)
Voucher (free)	209 (62.8)
Type of IUCD	
Multiload	255 (76.6)
Copper-T	78 (23.4)
Status of contraception before IUCD insertion	
Using a contraceptive method	113 (33.9)
Not using any method	220 (66.1)
Experience of side effects after IUCD insertion	
No	81 (24.3)
Yes	252 (75.7)

Number of cases	*N* = 333

**Table 2:** Method switching behavior among women who had IUCD removal.

Contraceptive status after IUCD removal	Contraceptive status before IUCD insertion			Overall switching after IUCD removal
	Nonuser *n* (%)	Short-term^1^ *n* (%)	Traditional^2^ *n* (%)	
Nonuser	100 (45.2)	30 (30.6)	3 (21.4)	133 (39.9)
Short-term^1^	64 (29.0)	44 (44.9)	3 (21.4)	111 (33.3)
Permanent^2^	18 (8.1)	5 (5.1)	0 (0.0)	23 (3.9)
Traditional^3^	39 (17.6)	19 (19.4)	8 (57.1)	66 (19.8)
Total	**221 (100.0)**	**98 (100.0)**	**14 (100.0)**	**333 (100.0)**

^1^Condom, oral pill, and injection.

^2^Female sterilization.

^3^Withdrawal and periodic abstinence.

**Table 3:** Unadjusted odds ratios of method switching versus method stopping, according to selected sociodemographic and reproductive health risk factors.

Characteristics	Method switched		
	*N*	*n* (%)	OR (95% C.I.)
Geographic region			
Northern Punjab	124	58 (46.8)	1
Sindh	59	30 (50.8)	1.17 (0.63–2.18)
Southern Punjab	150	112 (74.7)	3.35 (2.01–5.58)^*∗∗*^
Age of women			
≤25 years	21	11 (52.4)	1
>25–≤35 years	218	136 (62.4)	1.50 (0.61–3.70)
>35–49 years	94	53 (56.4)	1.17 (0.45–3.03)
Women education			
No formal education	207	119 (57.5)	1
Primary	72	42 (58.3)	1.03 (0.60–1.78)
≥Secondary	54	39 (72.2)	1.92 (0.99–3.70)
Number of children			
1-2	47	23 (48.9)	1
3-4	120	78 (65.0)	1.93 (0.97–3.84)
5+	166	99 (59.6)	1.54 (0.80–2.95)
Type of client			
Voucher (free)	209	117 (56.0)	1
Referral (paid out of pocket)	124	83 (66.9)	1.59 (1.00–2.52)^*∗*^
Type of IUCD			
Copper-T	78	36 (46.2)	1
Multiload	255	164 (64.3)	2.10 (1.25–3.51)^*∗∗*^
Status of contraception before IUCD insertion			
Not using any method	220	120 (54.5)	1
Using a contraceptive method	113	80 (70.8)	2.02 (1.24–3.27)^*∗∗*^
Reason for choosing IUCD			
Encouraged by FWM	148	88 (59.5)	1
Any other reason	185	112 (60.5)	1.04 (0.67–1.62)
Meeting with community after IUCD insertion			
No	99	39 (39.4)	1
Yes	234	161 (68.8)	3.39 (2.08–5.53)^*∗∗∗*^
Reason for IUCD discontinuation			
Nonhealth related	85	46 (54.1)	1
Method related side effects	248	154 (62.1)	1.39 (0.84–2.23)
Place of IUCD removal			
Government clinic	23	12 (52.2)	1
Private clinic	65	45 (69.2)	2.06 (0.78–5.46)
Expulsion	24	13 (54.2)	1.08 (0.34–3.41)
Suraj centre	221	130 (58.8)	1.30 (0.55–3.10)
Duration of IUCD use before discontinuation			
>6 to 24	165	83 (50.3)	1
>3 to 6	73	47 (64.4)	1.79 (1.01–3.15)^*∗*^
≤3 months	95	70 (73.7)	2.77 (1.60–4.80)^*∗∗∗*^
Time travel for removal services			
Less than 1 hour	305	183 (60.0)	1
≥1 hour	28	17 (60.7)	1.03 (0.46–2.28)
Satisfaction with IUCD services			
Satisfied or very satisfied	173	90 (52.0)	1
Neutral or unsatisfied	160	110 (68.8)	2.03 (1.29–3.18)^*∗∗*^
Would use IUCD in future, if needed			
Yes, readily	119	60 (50.4)	1
No or not sure	214	140 (70.0)	1.86 (1.18–2.93)^*∗∗*^
Would recommend IUCD to friend			
Yes	277	168 (60.6)	1
No	56	32 (57.1)	0.87 (0.48–1.55)

*P* value: ^*∗*^ *P* < 0.05,  ^*∗∗*^ *P* < 0.01, and  ^*∗∗∗*^ *P* < 0.001.

**Table 4:** Adjusted odds ratios of method switching versus method stopping, according to selected sociodemographic and reproductive health risk factors.

Characteristics	Method switched	
	AOR	(95% C.I.)
Region		
Northern Punjab	1	
Sindh	1.06	0.52–2.14
Southern Punjab	3.41	1.80–6.46^*∗∗∗*^
Type of client		
Voucher (free)	1	
Referral (paid out of pocket)	2.01	1.18–3.43^*∗*^
Status of contraception before IUCD insertion		
Not using any method	1	
Using a contraceptive method	2.26	1.31–3.87^*∗∗*^
Meeting with community health worker after IUCD insertion		
No	1	1
Yes	2.00	1.11–3.60^*∗*^
Satisfaction with IUCD related services		
Satisfied or very satisfied	1	1
Neutral or unsatisfied	1.72	1.04–2.82^*∗*^

*P* value: ^*∗*^ *P* < 0.05,  ^*∗∗*^ *P* < 0.01, and  ^*∗∗∗*^ *P* < 0.001.
